# Antimicrobial and Antifungal Activities of Terpene-Derived Palladium Complexes

**DOI:** 10.3390/antibiotics9050277

**Published:** 2020-05-25

**Authors:** Olga Zalevskaya, Yana Gur’eva, Aleksandr Kutchin, Karl A. Hansford

**Affiliations:** 1 Institute of Chemistry, FRC “Komi Scientific Centre”, Ural Branch of the Russian Academy of Sciences, ul. Pervomaiskaya 48, 167000 Syktyvkar, Russia; jana.aleksandrovna@yandex.ru (Y.G.); kutchin-av@mail.ru (A.K.); 2 Community for Open Antimicrobial Drug Discovery (CO-ADD), Centre for Superbug Solutions, Institute for Molecular Bioscience, The University of Queensland, Brisbane, QLD 4072, Australia; k.hansford@imb.uq.edu.au

**Keywords:** Pd(II) complexes, terpene ligands, antimicrobial, antifungal activities

## Abstract

In an era of multidrug-resistant bacterial infections overshadowed by a lack of innovation in the antimicrobial drug development pipeline, there has been a resurgence in multidisciplinary approaches aimed at tackling this global health problem. One such approach is to use metal complexes as a framework for new antimicrobials. Indeed, in this context, bismuth-, silver- and gold-derived compounds in particular have displayed demonstrable antimicrobial activity. In this work, we discuss the antimicrobial and antifungal activities of terpene-derived chiral palladium complexes against *Staphylococcus aureus*, *Pseudomonas aeruginosa*, *Klebsiella pneumoniae*, *Acinetobacter baumannii*, *Escherichia coli*, *Candida albicans*, and *Cryptococcus neoformans*. It was established that all studied coordination compounds of palladium were highly active antifungal drugs. In contrast, the subset of palladacycles possessing a palladium–carbon bond were only active against the Gram-positive bacterium *Staphylococcus aureus*. All compounds were inactive against the Gram-negative bacteria tested.

## 1. Introduction

Today, the number of metal-containing pharmacological preparations used in clinical practice is in the hundreds [1,2]. These are diagnostic substances and therapeutic drugs. Although anticancer agents have featured prominently, the concept of metal-derived compounds as antimicrobial agents is emerging [3,4]. Historically, preparations derived from silver [5,6], gold [7,8], bismuth [9], and more recently gallium [10,11] and ruthenium [12,13], have received attention. Specific examples of metallo-complexes demonstrating antimicrobial activity include N-heterocyclic carbene (NHS) complexes of silver and gold, auranofin (a clinically approved antirheumatic drug), colloidal bismuth citrate (CBS, a clinically approved antiulcer drug), and gallium nitrate. On the other hand, although palladium remains less explored in this context, there appears to be growing interest, with a steady number of studies published over the years [14,15,16,17,18,19,20]. Palladium palladacycles have historically garnered attention as anticancer agents due to their potential for diverse coordination geometry and cognate anticancer mechanisms distinct from those of platinum-based therapies such as *cis*-platin [20]. Although the mechanistic rationale of bacterial killing by metal-based drugs is often unclear, advances have been made to suggest that distinct molecular targets do exist, e.g., CBS irreversibly inhibits metallo-β-lactamases. Further mechanistic details for a range of metals have been summarized by Frei in this Special Issue [3]. 

The growing number of multidrug-resistant (MDR) microbes is a serious health threat to modern antimicrobial therapy, with a lack of technical innovation hampering the development of new drugs to address rising rates of resistance [21,22]. This has led to a resurgence in nontraditional approaches [3,23] to develop antimicrobial therapies that do not fit within the traditional paradigm of direct-acting small molecule drugs. Similarly, the Community for Open Antimicrobial Drug Discovery (CO-ADD) initiative focused on screening globally sourced novel chemical entities has paved the way for many researchers to become actively involved in antimicrobial research [24,25]. A recent review [4] reporting the evaluation of 906 metal-containing compounds screened for antimicrobial activity by CO-ADD demonstrated that metal-bearing compounds possessed a significantly higher hit rate (9.9%) when compared to non-metal based organic molecules (0.87%), with certain metals outperforming others. This observation suggests that bacterial susceptibility to chemotherapeutic metallo-complexes is significantly influenced by the nature of the coordinated metal ion and its associated coordination sphere. Indeed, in most of the aforementioned studies [14,15,16,17,18,19,20,21], it was found that palladium-derived complexes exhibited significantly higher antibacterial and antifungal activity compared to the corresponding free organic ligands.

In this work we report the antimicrobial activity and mammalian cellular toxicity of a series of chiral palladium-terpene complexes **1**–**12** against five types of bacteria, namely *Staphylococcus aureus, Pseudomonas aeruginosa*, *Klebsiella pneumoniae*, *Acinetobacter baumannii*, *Escherichia coli*, and two types of fungi (yeasts), namely *Candida albicans* and *Cryptococcus neoformans*. These ESKAPE pathogens (*Enterococcus* species, *Staphylococcus aureus*, *Klebsiella pneumoniae*, *Acinetobacter baumannii*, *Pseudomonas aeruginosa*, *Enterobacter* species), identified by the World Health Organization (WHO) as serious threats to human health, are the root cause of many drug-resistant infections [22,26,27]. 

## 2. Results and Discussion

### 2.1. Inhibition Agents

**Figure 1:**
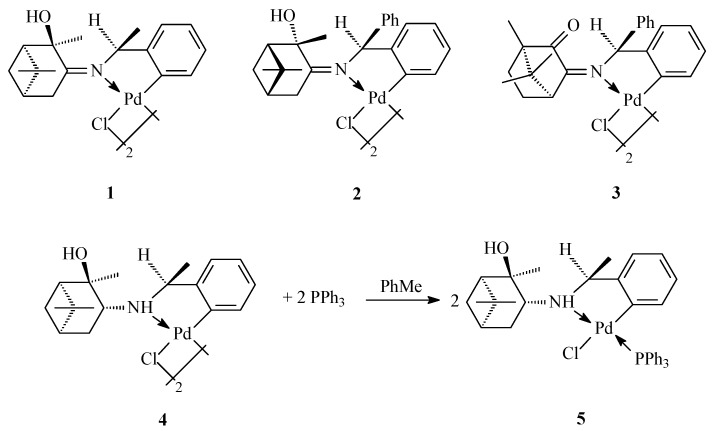
Cyclopalladated benzylamine derivatives.

**Figure 2:**
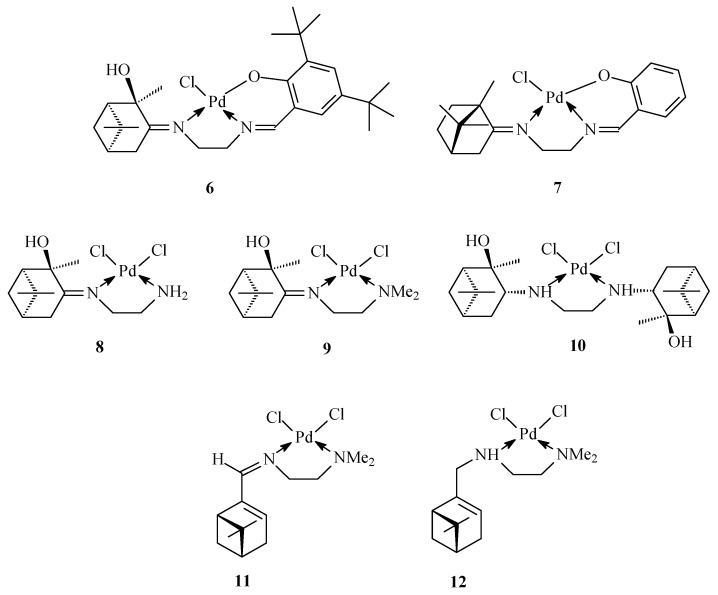
Palladium complexes with terpene derivatives of ethylenediamine.

The three types of chiral palladium complexes studied in the present work were derived from the natural terpenoids (−)-α-pinene, (+)-α-pinene, (−)-camphor, and (+)-camphor, with the *N*-donor atom in each complex originating from (−)-(s)-α-methylbenzylamine (compounds **1**, **4**, and **5**), benzhydrylamine (compounds **2** and **3**), or ethylenediamine (compounds **6**–**12**) (Figure 1 and Figure 2). Chiral metal complexes with terpene ligands are actively studied in various asymmetric transformations [28]. Complexes **1**–**4** are palladacycles containing a carbon–palladium bond obtained by direct cyclopalladation of the benzylamine moiety (Figure 1). Binuclear complexes of this type can be readily transformed into mononuclear mixed ligand derivatives to generate extensive libraries of organometallic complexes (e.g., compound **5**), with ligand diversity potentially derived from a diverse pool of pharmacologically relevant substituents, such as amino acids and heterocycles [29]. 

Ethylenediamine (EDA) derivatives have been successfully used as ligands for the synthesis of metal chelates with broad applicability, as exemplified by the complexes **6**–**12** (Figure 2). Compounds **6** and **7** belong to the Salen type complexes with tridentate *N*,*N*,*O*-donor ligands, whereas compounds **8**–**12** are palladium chelate complexes with bidentate 1,2-diaminoligands. 

### 2.2. Antimicrobial Activity and Mammalian Cellular Toxicity 

The antimicrobial activity of palladium complexes **1**–**12** was determined against selected reference strains of ESKAPE [26] bacteria - *E. coli*, *S. aureus* (MRSA), *K. pneumoniae*, *A. baumannii*, *P. aeruginosa*. No activity was observed against the Gram-negative pathogens; data for MRSA is presented in Table 1. The cellular toxicity of compounds **1**–**12** was also evaluated against human embryonic kidney cells (HEK293) and against human red blood cells (HRBC), as shown in Table 1.

Complexes **1**–**5**, possessing a carbon–palladium bond, exhibited varying degrees of inhibitory activity against MRSA, as measured by their minimum inhibitory concentrations (MIC 1—32 μg/mL), Table 1. Within the series **1**–**5**, palladacycle **2** displayed the greatest potency (MIC 1 μg/mL), and although accompanied by a degree of cytotoxicity (CC_50_ = 12.4 μg/mL) and haemolytic activity (HC_10_ = 1.83 μg/mL), it possessed the most promising selectivity index (SI) as determined by the ratio of CC_50_/MIC (12.4) and HC_10_/MIC (1.8), Table 2. The imine-linked compound **1**, displaying eight-fold greater potency than its amine-linked counterpart **4** (MIC 4 and 32 μg/mL, respectively), was also characterized by an approximately six-fold improved SI against both HEK293 and HRBC relative to **4**, suggesting that the pinan imine linkage may confer additional antimicrobial benefits with minimally associated toxicity. On the other hand, the transformation of binuclear palladacycle **4** into a mononuclear complex **5**, containing an additional triphenylphosphine ligand, did not offer much improvement in activity against *S. aureus* (MIC 16 μg/mL) nor associated SI, suggesting scope for further improvement. 

In contrast, the bidentate compounds **7**–**12**, lacking a direct C-Pd bond, were inactive against *S. aureus* at the highest concentration tested (MIC > 32 μg/mL), and were generally non-cytotoxic and non-hemolytic, suggesting some link between cellular toxicity and general Gram-positive antibacterial activity. The bidentate analog **6** was exceptional in this instance, displaying activity against MRSA (MIC 8 μg/mL), albeit with associated levels of toxicity (CC_50_/MIC = 0.6; HC_10_/MIC ~1), presumably due to the additional lipophilicity conferred by the *tert*-butyl groups.

Interestingly, despite the selective Gram-positive activity of compounds **1**–**12**, with their associated antifungal activity (see Section 3.4), compounds **1**–**12** were devoid of activity against the panel of Gram-negative pathogens (MIC > 32 μg/mL), highlighting the inherent difficulty of overcoming the permeability barrier of Gram-negative organisms due to potentially limited cellular entry and/or efflux. Notably, a similar trend was observed for most of the metal-containing compounds screened for antimicrobial activity by CO-ADD, whereby a hit rate of 1.5% was observed from a pool of 906 compounds [4]. 

Taken together, the results herein suggest that the killing effect of compounds **1**–**6** against *S. aureus* may be mediated in part by non-specific membrane disruption, leading to the observed cytotoxicity against HEK293 cells and haemolytic activity against HRBC. In a recent review [20], the cytotoxicity profiles of a variety of *N*,*C*-, *N*,*N*-, *N*,*S*-, and *N*,*O*-donor chelating ligand complexes against both cancerous and non-cancerous cell lines were reviewed, highlighting the potential for palladium complexes to possess promising therapeutic indices contingent upon ligand design and coordination geometry. 

### 2.3. Antifungal Activity and Mammalian Cellular Toxicity

In contrast to their antibacterial activity profiles, all of the studied palladium complexes **1**–**12** were active against both *C. albicans* and *C. neoformans var grubii*, (MIC ≤ 0.25—16 μg/mL), Table 1. Bidentate imine analog **11**, derived from α-pinene, displayed the greatest potency within the series (MIC ≤ 0.25 μg/mL against both species). Its amine-linked derivative **12**, although equipotent against *C. neoformans var grubii* (MIC ≤ 0.25 μg/mL) was greater than eight-fold less active against *C. albicans* (MIC 2 μg/mL), consistent with the activity trend observed against *S. aureus* between the matched imine- and amine-linked structural analogs **1** and **4**. Nonetheless, across the compound series, compound **12** displayed the least toxicity (SI > 128) against HEK293 and HRBC with respect to *C. neoformans var grubii*, suggesting that antimicrobial activity and general cytotoxicity can be uncoupled, Table 2. The bidentate imine-linked analog **9**, derived instead from hydroxypinanone, displayed reduced potency against both *C. albicans* (MIC 16 μg/mL) and *C. neoformans var grubii* (MIC 2 μg/mL) compared to α-pinene-derived **11**, suggesting modulation of antifungal activity is possible contingent upon ligand design. Their relative SI could not be compared due to non-unity CC_50_ and HC_10_ values (> 32 μg/mL). 

The influence of *N*,*N*-dimethyl substitution of the EDA ligand in **9** was probed by comparing the activity of related analog **8**, possessing a primary amine; both compounds displayed similar activity profiles (*C. albicans*: MIC 8—16 μg/mL; *C. neoformans var grubii*: MIC 2—4 μg/mL). On the other hand, substitution of both *N*-donor atoms of the EDA ligand with hydroxypinanone leading to compound **10** did not lead to improvements in activity against either yeast strain relative to compound **8** (possessing a single hydroxypinanone ligand), although increased cytotoxicity against HEK293 was observed for compound **10** (SI = 8). Lastly, compounds **6** and **7**, characterised by a tridentate arrangement derived from a combination of terpene- and salen-type ligands, respectively, exhibited moderate potency against both strains (MIC 4—16 μg/mL) with no apparent potency benefit over analogs **8**–**10**. However, compound **6** possessed distinctly greater cellular toxicity against HEK293 and HRBC (SI ~1–2) relative to compound **7** (SI > 8), likely due to its increased lipophilicity. 

Overall, the carbon-linked palladacycles **1**–**5**, possessing Gram-positive antibacterial activity, displayed greater potency against both yeast strains compared to compounds **6**–**10**, which lack a direct C-Pd bond. Notably, analogs **2** and **4**, possessing relatively potent MICs against both yeast strains (MIC ≤ 0.25—1 μg/mL), concomitantly displayed divergent potencies against *S. aureus* (MIC 1 and 32 μg/mL, respectively), suggesting independent mechanisms of antifungal and antibacterial activity. Both compounds also displayed promising SI against HEK293 relative to *C. neoformans var grubii* MICs (SI > 50), as shown in Table 2. Notably, compound **2** also possessed the best SI against HEK293 relative to *S. aureus* (SI = 12.4).

## 3. Materials and Methods

### 3.1. Synthesis of Complexes 

The synthesis, physicochemical and spectral characteristics of the complexes **1**–**4**, **6**–**12** have been reported previously [30,31,32,33,34,35,36]. The synthesis of the new complex **5** was carried out according to known procedures [31]. The NMR experiments were carried out using a Bruker AVANCE-II-300 spectrometer operating at 300.17 MHz for ^1^H and 75.48 MHz for ^13^C. Chemical shifts (δ) are reported in ppm relative to the residual solvent peak or internal standard (tetramethylsilane), and coupling constants (*J*) are reported in hertz (Hz). FT-IR spectra were recorded in the 200–4000 cm^−1^ region with a Shimadzu FT-IR 8400 spectrophotometer. Melting points were determined in open capillary tubes with an Electrothermal-9200 melting point device. Elemental analyses were performed by using EA 1110 CHNS-O apparatus. The optical rotations were measured at λ 589 nm on an Optical Activity PolAAr 3001 automated digital polarimeter.

(+)-trans-Chloro{(1R,2R,3R,5R)-3-((S)-1′-phenylethylamino)-2,6,6-trimethylbicyclo[3.1.1]heptane-2-ol-C,N}(triphenylphosphine)palladium(II) (**5**): white powder, yield 92%, m.p. 153–154 °C (with decomp.), [α]*_D_*+46.5 (*c* 0.2, CHCl_3_). Elemental analysis calculated. (%) for C_36_H_41_OPNClPd: C 64.0, H 2.5, N 6.7; found: C 63.9, H 2.1, N 6.1. IR ν KBr (cm^−1^): 3430 (OH), 3359 (NH). NMR ^1^H (CDCl_3_, δ/ppm J/Hz): 0.88 (s, 3H, CH_3_-9), 1.21 (s, 3H, CH_3_-8), 1.48 (m, 1H, H_α_-4), 1.52 (s, 3H, CH_3_-10), 1.53 (m, 1H, H_α_-7), 1.73 (m, 1H, H-5), 1.87 (d, 3H, CH_3_-12, *J* = 6), 1.96 (m, 1H, H_β_-7), 1.99 (m, 1H, H_β_-4), 4.09 (m, 1H, H-3), 4.38 (m, 1H, H-11), 4.59 (s, 1H, OH), 5.12 (br.s., 1H, NH), 6.19 (dd, 1H, H-15, *J* = 7.2, 7.2), 6.23 (dd, 1H, H-16, *J* = 7.2, 7.2), 6.71 (dd, 1H, H-17, *J* = 7.2, 6.7), 6.96 (d, 1H, H-18, *J* = 6.7), 7.41–7.64 (m, 15H, PPh_3_). NMR ^13^C (CDCl_3_, δ/ppm): 23.02 (C(8)), 24.12 (C(7)), 24.59 (C(10)), 27.20 (C(12)), 28.15 (C(8)), 31.60 (C(4)), 39.67 (C(6)), 40.46 (C(5)), 54.74 (C(1)), 63.42 (C(11)), 64.94 (C(3)), 76.70 (C(2)), 120.72 (C(18)), 124.19 (C(17)), 124.86 (C(16)), 128.07 d (6C, P-Ph*_m_*, *J* = 12), 130.70 d (3C, P-Ph*_p_*, *J* = 2), 131.36 d (C_Ar_-P, *J* = 49), 135.18 d (6C, P-Ph*_o_*, *J* = 12), 137.89 d (C-15, *J*_C-P_ = 8), 148.00 (C-13), 159.54 (C-14). NMR ^31^P (CDCl_3_): 59.0 ppm. 

### 3.2. Compound Preparation

All screened compounds were > 95% purity as determined by NMR. Dry compounds were dissolved in dimethyl sulfoxide (DMSO), and serially diluted 2-fold to produce an 8-point dose response with DMSO/water and then added into microtiter plates with a final DMSO concentration of 0.5%, providing a concentration range of 0.25 to 32 μg/mL. Screening against bacteria and yeasts for antimicrobial activity and toxicity against mammalian cells was performed in duplicate (*n* = 2).

### 3.3. Antibacterial Assays 

For the bacterial assays, each bacterial strain was cultured in Cation-Adjusted Mueller Hinton Broth (CAMHB; Bacto Laboratories 212322) at 37 °C overnight. Bacterial strains tested were methicillin resistant *S. aureus* (methicillin-resistant *Staphylococcus aureus* - MRSA) ATCC 43300, *E. coli* ATCC 25922, *K. pneumoniae* K6/ESBL ATCC 700603, *P. aeruginosa* ATCC 27853, *A. baumannii* ATCC 19606. A sample of each culture was then diluted 40-fold in fresh CAMHB and incubated at 37 °C for 1.5–3 h. The resultant mid-log phase cultures were diluted with CAMHB (CFU/mL measured by OD_600_), then added to each well of the compound-containing plates (384-well non-binding surface (NBS) plates; Corning CLS3640), giving a final cell density of 5 × 10^5^ CFU/mL and a total volume of 50 μL. Plates were covered and incubated at 37 °C for 18 h without shaking. Inhibition of bacterial growth was determined by measuring absorbance at 600 nm (OD_600_), using media only as negative control and bacteria without inhibitors as positive control. MIC values were determined as the lowest concentration at which the growth was inhibited by ≥ 80% (equivalent to no visible growth by eye). Colistin sulfate (Sigma Aldrich, Castle Hill, Australia C4461) and vancomycin HCl (Sigma Aldrich, 861987) were used as positive inhibitor controls on each plate for Gram-negative and Gram-positive bacteria, respectively.

### 3.4. Antifungal Assays 

For the fungal assays, yeast strains *C. neoformans var. grubii* H99 ATCC 208821 and *C. albicans* ATCC 90028 were cultured for 3 days on Yeast Extract-Peptone Dextrose (YPD; Becton Dickinson 242720) agar at 30 °C. A yeast suspension of 1 × 10^6^ to 5 × 10^6^ CFU/mL (as determined by OD_530_) was prepared from five colonies form the agar plates, and subsequently diluted with Yeast Nitrogen Base media (YNB; Becton Dickinson 233520), and added to each well of the compound containing plates (384-well plates, NBS; Corning CLS3640) giving a final cell density of 2.5 × 10^3^ CFU/mL and a total volume of 50 μL. Plates were covered and incubated at 35 °C for 36 h without shaking. Growth inhibition of *C. albicans* was determined measuring absorbance at 630 nm (OD_630_), while the growth inhibition of *C. neoformans* was determined measuring the difference in absorbance between 600 and 570 nm (OD_600-570_), after the addition of resazurin (0.001% final concentration; Sigma R7017) and incubation at 35 °C for 2 h, using media only as negative control and fungi without inhibitors as positive control. MIC values were determined as the lowest concentration at which the growth was inhibited at ≥ 80% (equivalent to no visible growth by eye). Fluconazole (Sigma Aldrich, F8929) was used as a positive inhibitor control on each plate for both strains.

### 3.5. Cytotoxicity Assays 

Cytotoxicity was assessed against human embryonic kidney cells (HEK293, ATCC CRL-1573), with data presented as CC_50_, corresponding to the concentration of the drug (μg/mL) at which 50% inhibition of cell growth is achieved. HEK293 cells were counted manually in a Neubauer hemocytometer and added to compound-containing plates (384-well plates, tissue culture treated (TC); Corning CLS3712) giving a final density of 5000 cells/well in a total volume of 50 μL, using Dulbecco’s Modified Eagle Medium (DMEM; Life Technologies 11995-073) with 10% Fetal Bovine Serum (FBS; GE SH30084.03). The cells were incubated together with the compounds for 20 h at 37 °C in 5% CO_2_. Cytotoxicity (or cell viability) was measured by fluorescence, ex: 560/10 nm, em: 590/10 nm (F560/590), after addition of 5 μL of 25 μg/mL resazurin (2.3 μg/mL final concentration; Sigma R7017) and after further incubation for 3 h at 37 °C in 5% CO_2_. Media only was used as negative control and cells without inhibitors as positive growth control. CC_50_ (concentration at 50% cytotoxicity) values were calculated by curve fitting the inhibition values vs. log(concentration) using a sigmoidal dose-response function, with variable fitting values for bottom, top and slope. Tamoxifen (Sigma Aldrich, Australia T5648) was used as positive inhibitor control on each plate. Selectivity indices (SI) were calculated by dividing the CC_50_ value (μg/mL) by the MIC value (μg/mL) for a given bacterial or fungal strain. 

### 3.6. Haemolysis Assays

Hemolytic activity was assessed against human red blood cells (HRBC), with data presented as HC_10_, corresponding to the concentration of the compound causing 10% hemolysis. Human whole blood (Australian Red Cross) was washed three times with 3 volumes of 0.9% NaCl and resuspended in a concentration of 0.5 × 10^8^ cells/mL, determined by manual cell count in a Neubauer hemocytometer. Washed cells were added to compound containing plates (384-well polypropylene plates (PP); Corning 3657) for a final volume of 50 μL. The plate was shaken with incubation for 1 h at 37 °C. After incubation, the plates were centrifuged at 1000× g for 10 min to pellet cells and debris. Supernatant (25 μL) was then transferred to reading plates (384-well, polystyrene plated (PS), Corning CLS3680), with hemolysis determined by measuring the supernatant absorbance at 405 mm (OD405), using cells without inhibitors as negative control and cells with 1% Triton X-100 (Sigma T8787) as positive control. HC_10_ and HC_50_ (concentration at 10% and 50% hemolysis, respectively) were calculated by curve fitting the inhibition values vs. log(concentration) using a sigmoidal dose-response function with variable fitting values for top, bottom and slope. Melittin (Sigma M2272) was used as positive hemolytic control on each plate. Human blood was sourced from the Australian Red Cross Blood Service. Selectivity indices (SI) were calculated by dividing the HC_10_ value (μg/mL) by the MIC value (μg/mL) for a given bacterial or fungal strain. 

The use of human blood (sourced from the Australian Red Cross Blood Service) for hemolysis assays was approved by The University of Queensland Institutional Human Research Ethics Committee, Approval Number 2014000031.

## 4. Conclusions

A series of coordination compounds **1**–**12**, derived from palladium with terpene ligands, were studied as antibacterial and antifungal agents. The complexes were found to be broadly active against *C. albicans* and *C. neoformans var grubii*, whereas in the context of bacteria, they possessed activity toward MRSA but were inactive toward Gram-negative pathogens. Compound **2** displayed potent activity against all three pathogens (MIC ≤ 0.25—1 μg/mL) and possessed the best SI with respect to *S. aureus* (12.4 and 1.8 against HEK293 and HRBC, respectively), warranting further investigation. Compound **12** was inactive against *S. aureus* but exhibited promising activity against *C. albicans* and *C. neoformans var grubii* and possessed exceptional SI with respect to *C. neoformans var grubii* (> 128 against HEK293 and HRBC), highlighting the need for further microbiological evaluation. The results herein suggest that palladium complexes may represent promising starting points for antimicrobial development, whereupon the manipulation of various ligand properties and coordination geometries may pave the way for the synthesis of a diverse array of palladium complexes with desired antimicrobial characteristics devoid of mammalian cellular toxicity.

## Figures and Tables

**Table 1:** Minimum inhibitory concentration (MIC) of Pd(II) complexes against *S. aureus* (MRSA), *C. albicans* and *C. neoformans var grubii H99* (μg/mL), CC_50_ (concentration (μg/mL) of 50% cytotoxicity) against HEK293 and HC_10_ (concentration (μg/mL) of 10% haemolysis of human red blood cells).

Compound	*S. aureus* ATCC 43300	*C. albicans* ATCC 90028	*C. neoformans* ATCC 208821	CC_50_	HC_10_
**1**	4	1	0.5	11.9	3.9
**2**	1	0.5	≤0.25	12.4	1.8
**3**	16	2	0.5	6.0	>32
**4**	32	1	≤0.25	16.6	5.6
**5**	16	2	1	2.9	5.6
**6**	8	8	4	4.5	7.8
**7**	>32	16	4	>32	>32
**8**	>32	8	4	>32	6.9
**9**	>32	16	2	>32	>32
**10**	>32	4	4	>32	4.6
**11**	>32	≤0.25	≤0.25	10.8	>32
**12**	>32	2	≤0.25	>32	>32
**vancomycin**	1	--	--	--	--
**fluconazole**	--	0.125	8	--	--
**tamoxifen**	--	--	--	9	--
**melittin**	--	--	--	--	2.7

**Table 2:** Selectivity indices (SI) of compounds determined for *S. aureus* (MRSA) ATCC 43300 and *C. neoformans var grubii* H99 ATCC 208821, in relation to CC_50_ (concentration (μg/mL) of 50% cytotoxicity) against HEK293 and HC_10_ (concentration (μg/mL) of 10% haemolysis of human red blood cells).

	Selectivity Index (SI)			
Cmpd	*S. aureus* (MRSA)		*C. neoformans*	
	CC_50_/MIC	HC_10_/MIC	CC_50_/MIC	HC_10_/MIC
**1**	3.0	1.0	23.8	7.8
**2**	12.4	1.8	>49.6	>7.2
**3**	0.4	>2	12.0	>64
**4**	0.5	0.2	>66.4	>22.4
**5**	0.2	0.4	2.9	5.6
**6**	0.6	1.0	1.1	2.0
**7**	N/A	N/A	> 8	>8
**8**	N/A	≤0.2	> 8	1.7
**9**	N/A	N/A	> 16	>16
**10**	N/A	≤0.1	> 8	1.2
**11**	≤0.3	N/A	> 43.2	>128
**12**	N/A	N/A	> 128	>128

N/A, selectivity index could not be calculated as the compound was inactive against the strain of interest or did not possess toxicity.
